# Differentiation of CD166-positive hPSC-derived lung progenitors into airway epithelial cells

**DOI:** 10.1242/bio.061729

**Published:** 2024-10-10

**Authors:** Kim Jee Goh, Hao Lu, Ee Kim Tan, Zhao Yong Lee, Amanda Wong, Thai Tran, N. Ray Dunn, Sudipto Roy

**Affiliations:** ^1^ Skin Research Institute of Singapore, Clinical Sciences Building, 11 Mandalay Road #17-01, Singapore 308232, Singapore; ^2^ Institute of Molecular and Cell Biology (IMCB), Agency for Science, Technology and Research (A*STAR), 61 Biopolis Drive, Singapore 138673, Singapore; ^3^ Lee Kong Chian School of Medicine, Nanyang Technological University, Clinical Sciences Building, 11 Mandalay Road, Singapore 308232, Singapore; ^4^ Infectious Diseases Translational Research Programme, Department of Medicine, Yong Loo Lin School of Medicine, National University of Singapore, 21 Lower Kent Ridge Road, Singapore 119077, Singapore; ^5^ Department of Physiology, Yong Loo Lin School of Medicine, National University of Singapore, 2 Medical Drive, MD9, Singapore 117593, Singapore; ^6^ Department of Paediatrics, Yong Loo Lin School of Medicine, National University of Singapore, 1E Kent Ridge Road, Singapore 119288, Singapore

**Keywords:** Human pluripotent stem cells, Directed differentiation, CD166, Proximal lung airway, Organoids, Air-liquid interface, Multiciliated cells, Influenza

## Abstract

The generation of lung epithelial cells through the directed differentiation of human pluripotent stem cells (hPSCs) *in vitro* provides a platform to model both embryonic lung development and adult airway disease. Here, we describe a robust differentiation protocol that closely recapitulates human embryonic lung development. Differentiating cells progress through obligate intermediate stages, beginning with definitive endoderm formation and then patterning into anterior foregut endoderm that yields lung progenitors (LPs) with extended culture. These LPs can be purified using the cell surface marker CD166 (also known as ALCAM), and further matured into proximal airway epithelial cells including basal cells, secretory cells and multiciliated cells using either an organoid platform or culture at the air-liquid interface (ALI). We additionally demonstrate that these hPSC-derived airway epithelial cells can be used to model Influenza A infection. Collectively, our results underscore the utility of CD166 expression for the efficient enrichment of LPs from heterogenous differentiation cultures and the ability of these isolated cells to mature into more specialized, physiologically relevant proximal lung cell types.

## Teaser

**Summary:** Human pluripotent stem cell (hPSC)-derived lung progenitors were isolated using CD166 and differentiated into airway epithelial cells. These hPSC-derived airway epithelial cells were then shown to be useful for studying respiratory infections, such as Influenza A.

## INTRODUCTION

The lungs have evolved an extensive branching network of epithelial tubes that facilitate gas exchange. This network consists of the conducting airway, bronchioles and distal alveolar sacs that maximize the surface area for efficient gas exchange. Individuals with congenital defects in lung development or those with diseases that afflict the lungs and interfere with their function suffer severe or fatal consequences (Collaborators, 2020). The development of novel treatment modalities for such patients involving cell therapy and regenerative medicine requires a deep understanding of how the diverse proximal and distal lung cell types, comprising the normal lung, emerge during human embryogenesis.

The availability and utility of animal models, notably the mouse, have led to tremendous progress in understanding lung development in health and disease. However, physiological and biological differences have also become increasingly apparent between human and non-human animal species (Kho et al., 2010; Nikolić et al., 2017). For example, murine models fail to recapitulate the severe phenotypes observed in the pancreas and the lungs of patients with cystic fibrosis (Semaniakou et al., 2018). Given this shortfall, studies that rely solely on animal models are limited in their applicability to humans. Thus, there is tremendous interest in developing human *in vitro* model systems to complement and enhance data obtained from animal models. Such *in vitro* platforms also offer other advantages, such as the ability to tightly control the experimental environment when investigating complex biological processes, alongside their reproducibility and potential for scalability.

To date, several *in vitro* human lung models have been described. These include primary cells from donors, immortalized cell lines and human pluripotent stem cell (hPSC)-derived lung epithelial cells. Each of these *in vitro* model systems is associated with its own set of strengths and limitations. Cells derived from primary human lung tissue have the advantage of being more biologically relevant and patient-specific. However, primary lung tissue from patients is difficult to procure, and such cells cannot be maintained over extended passaging due to the eventual loss of proliferative and/or differentiation potential, despite numerous efforts to extend their lifespan and multipotency (Gentzsch et al., 2017; Liu et al., 2012; Mou et al., 2016). There is also the major hurdle of extensive donor heterogeneity, rendering it difficult to deploy these cells in high-throughput screens and to compare results generated in different laboratories. These issues can be overcome by using immortalized cell lines generated through the overexpression of viral oncogenes, cell cycle proteins, or telomerase (hTERT) (Fulcher et al., 2009; Gruenert et al., 2004; Ramirez et al., 2004). However, immortalization or transformation has been reported to compromise multipotency, functionality and genetic stability (da Paula et al., 2005; Gruenert et al., 2004; Peters-Hall et al., 2018; Zabner et al., 2003).

hPSC-derived lung epithelial cells have garnered much attention in recent years as they may overcome these limitations. These cells, whether derived from human embryonic stem cells (hESC) or human induced pluripotent cells (hiPSC), provide reproducibility and scalability. Moreover, hPSCs can be combined with genome editing technologies to introduce or correct disease-causing mutations for disease modelling or (amenable) regenerative medicine applications (Jacob et al., 2017; McCauley et al., 2017). In recent years, several groups have published protocols for differentiating hPSC into lung epithelial cells (Dye et al., 2015; Gotoh et al., 2014; Huang et al., 2014; Jacob et al., 2017; Konishi et al., 2016; McCauley et al., 2017). Although there are differences among existing protocols, e.g. provision of growth factors, their concentration, the addition of small molecule inhibitors, media formulations, etc., all of them involve the stepwise directed differentiation of hPSCs into the definitive endoderm, followed by the anterior foregut endoderm and finally into lung progenitors, which can then be further matured into distal and/or proximal lung lineages (Dye et al., 2016; Ghaedi et al., 2015; Gomperts, 2014; Snoeck, 2015). hPSC-derived lung epithelial cells, however, are not without their limitations. Lung epithelial cells from differentiating hPSCs more closely resemble the fetal lung instead of the adult lung (Chen et al., 2017; Dye et al., 2015). Therefore, efforts continue to further elaborate the culture requirements and underlying molecular mechanisms that can promote the robust maturation of hPSC-derived lung epithelial cells *in vitro*.

In this study, we developed a modified stepwise directed differentiation protocol using the extensively studied hESC line, H9, to produce lung epithelial cells. Significantly, we show that the cell surface marker CD166 [also known as Activated Leukocyte Cell Adhesion Molecule (ALCAM)], which is highly expressed in the developing human lung at the pseudoglandular stage (weeks 7-16) (Soh et al., 2012), can be used to efficiently purify early lung progenitors (LP) from heterogeneous differentiation cultures. CD166+ cells can be further induced to form proximal lung epithelial cell types either as 3D organoids or cultures at the air-liquid interface (ALI). We additionally establish that these airway cells provide a robust, physiologically relevant *in vitro* platform to study influenza infection.

## RESULTS

### Characterization of hPSC-derived CD166+ lung progenitors

CD166 has been previously used as a marker of hPSC-derived LP cells, that when transplanted into immunocompromised mice with acute lung injury, differentiate into *distal* (alveolar) lung cell types (Soh et al., 2012). Whether CD166+ LP cells can differentiate into *proximal* airway epithelial cells remains unknown. To address this outstanding question, we first differentiated H9 cells for 15 days using established protocols, with minor modifications (McCauley et al., 2017; Vallier et al., 2009) (Fig. S1A). We next employed a commercially available phycoerythrin-conjugated anti-CD166 antibody to isolate early LPs from differentiation cultures. The percentage of CD166+ cells obtained after Fluorescent Activated Cell Sorting (FACS) was variable, ranging from 25% to over 90%, a result emphasizing the variability inherent in *in vitro* differentiation protocols (Fig. S1B,C). Quantitative real-time PCR (qPCR) revealed that the expression of a suite of cardinal early LP markers (*NKX2-1*, *SOX2*, *SOX9* and *FOXA2*) was significantly higher in the CD166+ cell fraction than in control (CD166-) (Hawkins et al., 2017) (Fig. S1D). Of note, we observed that the CD166+ cell fraction did not express higher levels of these markers when compared to the unsorted fraction, suggesting that CD166 might not be capturing all the lung progenitors from the differentiation cultures (Fig. S1D).

### Differentiation of CD166+ lung progenitors into proximal lung organoids

**Fig. 1.**
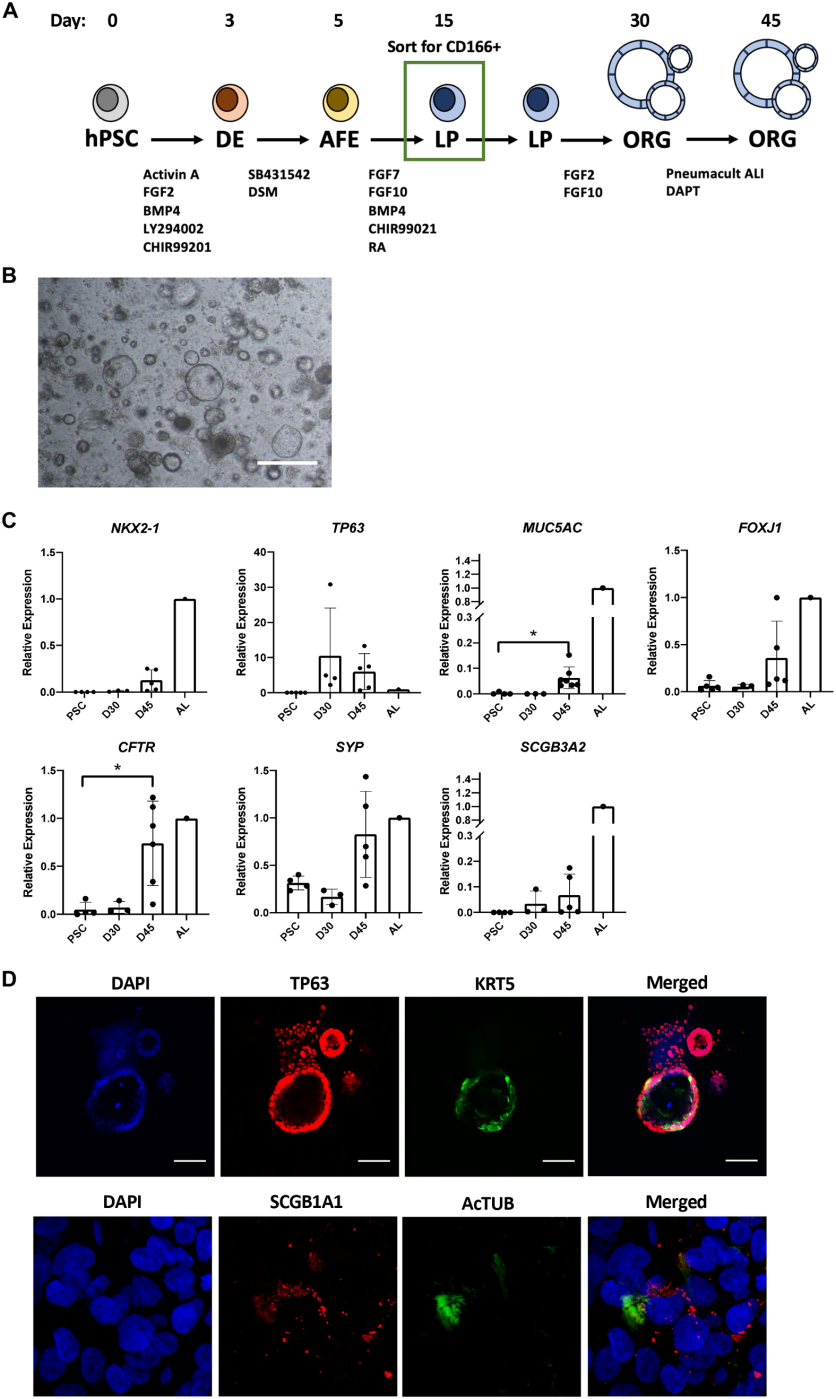
**CD166+ cells can be differentiated as proximal lung organoids.** (A) Schematic showing differentiation of hPSCs into lung progenitors and then into lung organoids. (B) Representative brightfield image of lung organoids on day 30. Scale bar: 200 µm. (C) qPCR analysis of proximal lung markers *NKX2-1*, *TP63*, *MUC5AC*, *FOXJ1*, *CFTR*, *SYP* and *SCGB3A2*. Data generated were from four independent experiments and are presented as means±s.d. *P<0.05. PSC: pluripotent stem cell; D (day), and AL (Adult Lung). (D) Representative immunostaining for basal cells (TP63, KRT5), multiciliated cells (FOXJ1, ACTUB) and secretory cells (MUC5AC). Scale bars: 100 µm. *n*=3.

hPSC-derived CD166+ LPs were next differentiated into proximal lung organoids (Fig. 1A). Sorted CD166+ cells formed organoids of various sizes in matrigel drops (Fig. 1B), and at Day 30, expressed markers characteristic of the proximal lung lineage such as *TP63*, *MUC5AC*, *FOXJ1*, *CFTR*, *SYP* and *SCGB3A2* (Fig. 1C). Expression of these markers increased when cultured for an additional 15 days in the presence of the NOTCH pathway inhibitor, DAPT (Fig. 1C). Although *NKX2-1* is known to be highly expressed in more distal lung lineages, some expression was also observed in the lung organoids (Fig. 1C). Immunostaining also demonstrated the presence of basal cells (TP63+ and KRT5+), secretory cells (SCGB1A1+) and multiciliated cells (AcTUB+) (Fig. 1D). Taken together, these data show that CD166+ LPs can be differentiated into proximal lung cell types.

### Differentiation of CD166+ lung progenitors into epithelial cells cultured at air-liquid interface (ALI)

Receptors for most respiratory viruses are located on the apical surface of airway epithelial cells. Most organoids are cultured in matrigel basement membrane matrix and are therefore oriented with the apical surface facing inwards, rendering their apical surface inaccessible to microbial pathogens and limiting their utility in modelling respiratory infection. Several methods have, however, recently been developed to study infection using organoids. For example, organoids can be sheared and plated onto transwells in a monolayer to expose the apical surface prior to pathogen inoculation (Aguilar et al., 2021). Alternatively pathogens can be directly microinjected into the organoid lumen, and techniques have been developed to generate ‘apical-out’ organoids (Aguilar et al., 2021).

**Fig. 2.**
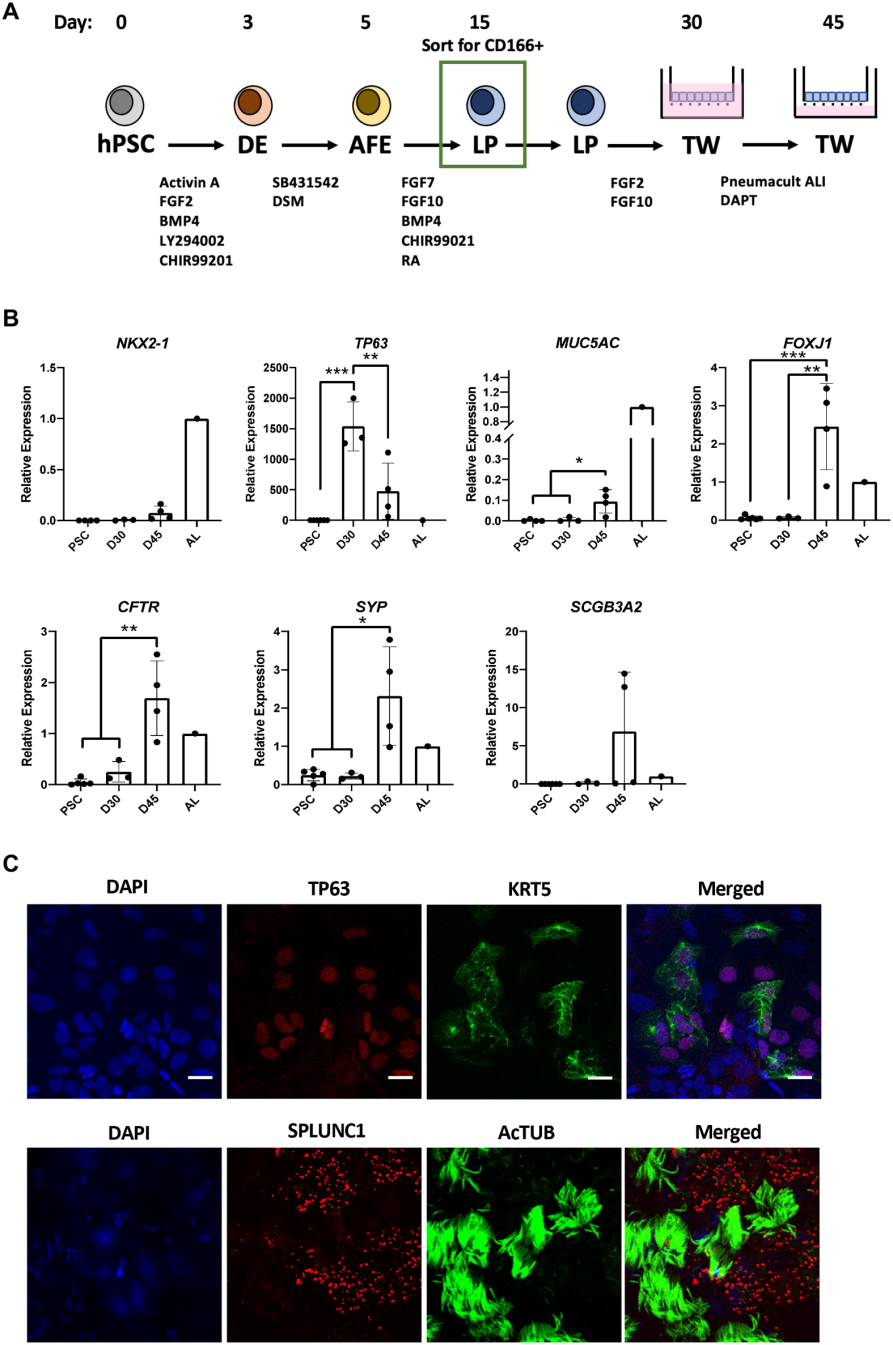
**CD166+ cells can be differentiated into airway epithelial cells on transwells.** (A) Schematic depicting differentiation of LPs on transwells for 15 days. Further differentiation and maturation were induced by culturing cells at an air-liquid interface for a further 15 days. (B) qPCR analysis of proximal lung markers NKX2-1, TP63, MUC5AC, FOXJ1, CFTR, SYP and SCGB3A2. Data from at least three independent experiments presented as means±s.d. *P<0.05, **P<0.01, ***P<0.001. (C) Immunostaining of markers associated with basal cells (TP63, KRT5), multiciliated cells (AcTUB) and secretory cells (SPLUNC1). Representative images shown. Scale bars: 5 µm. *n*=3.

To determine whether these hPSC-derived lung epithelial cells can serve as a model for infection by respiratory viruses, we differentiated hPSC-derived CD166+ lung progenitors in transwell monolayers (Fig. 2A). qPCR analysis of cells at Day 30 showed expression of *TP63* and low levels of *MUC5AC*, *FOXJ1*, *CFTR*, *SYP* and *SCGB3A2*. Expression of *MUC5AC*, *FOXJ1*, *CFTR*, *SYP* and *SCGB3A2* increased with ALI culture (Day 45), while expression of *TP63* decreased (Fig. 2B), suggesting that ALI culture and the addition of DAPT instigated further differentiation of the basal cells into mature proximal lung cell types. Low levels of *NKX2-1* expression were detected on both Day 30 and Day 45 (Fig. 2B). Immunostaining of Day 45 cultures indicated the presence of basal cells (TP63, KRT5), multiciliated cells (AcTUB) and secretory cells (SPLUNC1) in the transwell-ALI cultures (Fig. 2C). Thus, similar to the organoid model, CD166+ LPs can also be differentiated into proximal airway epithelial cells in transwell-ALI cultures.

### Modelling influenza A viral infection in hPSC-derived airway epithelial cells

The World Health Organization estimates the global burden of seasonal influenza at 3 to 5 million cases of severe illness and up to 650,000 deaths annually (2018). The rising number of drug-resistant influenza strains against both commercially available and novel antivirals signals a lack in competency to respond to impending influenza outbreaks (Kormuth and Lakdawala, 2020). Furthermore, animal model viral challenges often demand the adaptation of viruses to the host model. This potentially alters the inherent characteristics that facilitate viral infections in humans (Yan et al., 2016).

**Fig. 3.**
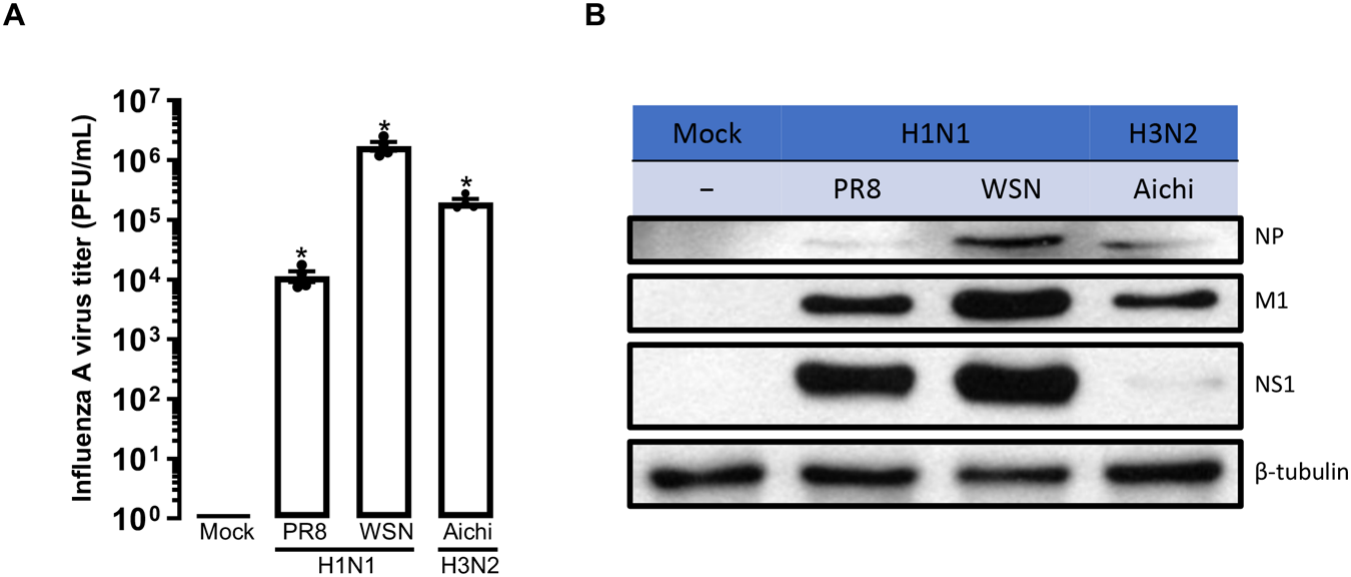
**hPSC-derived airway epithelial cells are susceptible to influenza infection.** (A) Virus plaque assay of influenza A virus samples [A/Puerto Rico/8/1934 (PR8), A/WSN/1933 (WSN), and A/Aichi/2/1968 (Aichi)] obtained from hPSC-derived airway epithelial cells. Cells were infected at a multiplicity of infection (MOI) of 0.1 or mock infected (Mock), with media containing trypsin-TPCK. Virus samples were collected 24 h post-infection (hpi). One-way ANOVA (repeated measures), multiple comparisons with Dunnett's correction, means±s.e.m., *P<0.05, technical replicates, *n*=4. (B) Representative western blot images showing nucleoprotein (NP), nonstructural protein 1 (NS1), matrix protein 1 (M1) and β-tubulin 24 hpi from influenza A virus infected hPSC-derived airway epithelial cells.

To identify key factors in the influenza life cycle, there is an imperative to establish an *in vitro* lung epithelial cell model that sufficiently recapitulates the viral pathophysiology of influenza infections. We demonstrate that our hPSC-derived airway epithelial cells can be infected by both H1N1 (A/Puerto Rico/8/1934 & A/WSN/1933) and H3N2 (A/Aichi/2/1968) strains, showing detectable virus titre (Fig. 3A) that was not strain-specific. Furthermore, we could show that influenza A viral proteins, nucleoprotein (NP), nonstructural protein 1 (NS1), and matrix protein 1 (M1) can be isolated from cellular lysates (Fig. 3B). This suggests that influenza A viruses are not only capable of entry into our hPSC-derived airway epithelial cells, but they are also capable of hijacking the host transcription and translational machinery for viral protein production. Taken together, these findings demonstrate that our ALI cultures are physiologically viable hosts for influenza A viral replication, serving as an alternative and representative *in vitro* model for influenza A virus infection studies.

## DISCUSSION

CD166 is a transmembrane glycoprotein belonging to the immunoglobulin superfamily that is involved in diverse cellular processes, including T-cell activation, haematopoiesis, trans-endothelial migration of neutrophils, angiogenesis, inflammation, and tumor growth and metastasis (Ofori-Acquah and King, 2008; Tachezy et al., 2012a,b; van Kilsdonk et al., 2008; Weidle et al., 2010). In addition, Soh et al. (2012) alongside other groups have previously used CD166 as a cell surface marker to isolate lung stem or progenitor populations (Hegab et al., 2012; Soh et al., 2012; Weeden et al., 2017). While these authors showed that isolated CD166+ cells can differentiate into distal lung lineages *in vitro* and *in vivo*, we show here that CD166+ cells can also differentiate into proximal lung lineages in the form of 3D organoids or 2D monolayer cultures at the ALI.

CD166+ cells isolated on Day 15 of differentiation expressed early LP markers such as *NKX2-1*, *FOXA2*, *SOX2* and *SOX9*. Although increased expression levels of proximal lung markers (*TP63, MUC5AC, FOXJ1, CFTR, SYP and SCGB3A2*) were observed in proximal lung organoids at the mRNA level, it was difficult to consistently detect the presence of multiciliated cells via immunostaining compared to cultures differentiated on transwells at the ALI. These results are consistent with the observations made by McCauley et al. (2017) whose differentiation protocol did not reliably generate multiciliated cells without the use of NOTCH pathway inhibitors or ALI culture (McCauley et al., 2017). Exposure of differentiating lung progenitors to air may be key to achieving optimal differentiation rates. Indeed, submersion and hypoxia have been reported to be refractory to human bronchial epithelial cell differentiation, particularly towards multiciliated cells (Gerovac et al., 2014). More comprehensive studies, comparing the differentiation efficiencies between 2D and 3D cultures, are therefore warranted. Additionally, it may be possible to improve differentiation rates of lung organoids through the supplementation of IL-6 or IL-13, which have been reported to promote differentiation into multiciliated cells and secretory cells, respectively (Gomperts, 2014; Tadokoro et al., 2014).

Lastly, we demonstrate the utility of hPSC-derived proximal lung epithelial cells on transwells at ALI for the investigation of the pathogenesis of respiratory viruses, specifically influenza viruses. hPSC-derived lung epithelial or progenitor cells have been previously used to study respiratory viruses (Chen et al., 2017; Ciancanelli et al., 2015; Huang et al., 2020; Jenny et al., 2015; Lim et al., 2019; Porotto et al., 2019; Yang et al., 2020). For example, Ciancanelli et al. (2015) and Lim et al. (2019) differentiated patient-derived iPSCs with mutations in *INTERFERON REGULATORY FACTOR 7 (IRF7)* and *TOLL-LIKE RECEPTOR 3 (TLR3)* to investigate and model influenza virus susceptibility (Ciancanelli et al., 2015; Lim et al., 2019). Both groups used a protocol that (1) does not involve the isolation of LPs prior to further maturation into lung epithelial cell types; (2) yields a mixture of proximal airway and distal alveolar lung epithelial cell types; and (3) involves differentiation under submerged culture conditions (Huang et al., 2014). In contrast to these two studies, our work involves the use of CD166 to isolate LPs and the (near) terminal differentiation of these cells into specifically proximal airway cell-types including basal, secretory and multiciliated cells. Isolation of LPs prior for further maturation is important as differentiation cultures are rarely homogeneous. In fact, Lim et al. (2019) noted that the percentage of NKX2-1+ lung epithelial cells at Day 55 varied widely (10.625 to 98.174%) between hiPSC lines and experiments (Lim et al., 2019). In both studies, NKX2-1 expression via immunofluorescence was used to characterize and identify lung epithelial cells. While NKX2-1 is an important lung marker, its expression has been reported to vary as lung development progresses, eventually becoming restricted to distal lung cell types (Stahlman et al., 1996). Ciancanelli et al. (2015) reported efficient replication of Influenza A virus in NKX2-1- cells (Ciancanelli et al., 2015). It is however unknown if these NKX2-1- cells are non-lung cell types or proximal airway cell types. We propose that purified hPSC-derived LPs cultured and terminally differentiated at ALI provide a better model system as it more closely resembles the human airway *in vivo*.

## MATERIALS AND METHODS

### Human pluripotent stem cell culture

H9 hESCs (WA09) were obtained from WiCell and were routinely cultured on Vitronectin-coated tissue culture dishes in Essential 8 (E8) medium (Life Technologies) in an incubator with 5% CO_2_ at 37°C. Cells were not recently authenticated but were regularly screened for mycoplasma contamination.

### Differentiation of hPSCs into lung progenitors

hPSCs were differentiated for 15 days into lung progenitors as described previously (48), with minor modifications. Briefly, on Day 0 (D0), CDM-PVA medium containing 100 ng/ml Activin A, 80 ng/ml FGF2, 10 μM LY294002, 3 μM CHIR99021, 10 ng/ml BMP4 was applied to the hPSCs. On D1, hPSCs were treated with 100 ng/ml Activin A, 80 ng/ml FGF2, 10 μM LY294002 and 10 ng/ml BMP4 in CDM-PVA medium. On D2, CDM-PVA medium with 100 ng/ml Activin A, 80 ng/ml FGF2, 1X B27, 1X NEAA in RPMI medium was added to the differentiation cultures. To promote anterior foregut formation, cells were grown in basal medium (1X B27, 1X N2, 1X Glutamax, 1 mM HEPES in DMEM F12 Advanced) containing 10 μM SB431542 and 2 μM Dorsomorphin (DSM) for 3 days. Finally, to differentiate AFE cells into lung progenitors, cells were cultured in basal medium supplemented with 3 μM CHIR, 10 ng/ml BMP4, 10 ng/ml FGF7, 10 ng/ml FGF10 and 50 nM RA for 9 days.

### Lung organoid culture

Lung organoids were generated as described previously (48) with minor modifications. Briefly, Day 15-sorted CD166+ cells were resuspended in basal medium containing 250 ng/ml FGF2, 100 ng/ml FGF10, 50 nM Dexamethasone (Sigma-Aldrich), 0.1 mM 8-Bromoadenosine 30,50-cyclic monophosphate sodium salt (cAMP, Sigma-Aldrich) and 0.1 mM 3-Isobutyl-1-methylxanthine (IBMX) (Sigma- Aldrich). The cell suspension and undiluted growth factor-reduced Matrigel (Corning) were mixed at a 1:1 ratio, after which 40 μl of this cell suspension was added to the middle of each well of a 24-well plate to create a Matrigel drop. The Matrigel drops were left to solidify in the incubator for at least 30 min before the medium was overlaid. Medium was supplemented with 10 μM Y-27632 for the first 24 h. Subsequently, the medium was refreshed every other day for 2 weeks (Day 30). From Day 31 to Day 45, PneumaCult ALI medium (STEMCELL Technologies) supplemented with 10 μM DAPT (Sigma-Aldrich), was overlaid on the Matrigel drops. This medium was refreshed every other day until Day 45.

### Fluorescence-Activated Cell Sorting (FACS)

Day 15 differentiated hPSCs were washed once with Dulbecco phosphate-buffered saline (DPBS) and then incubated with TryPLE express for 15-30 min at 37°C. DMEM Advanced F12 was used to dilute out the TrypLE express, and cells were collected into a pellet by centrifugation at 1200 rpm for 5 min. The cell pellet was resuspended in FACS buffer supplemented with 2% pen/strep and 10 μM Y-27632. 0.5 μl isotype control or 0.5 μl Human ALCAM/CD166 Phycoerythrin (PE)-conjugated antibody (Clone 105902) (R&D Systems) was added per 1 million cells in 100 μl. Cells were stained for 30 min in the dark at 4°C. Cells were collected into a pellet by centrifugation at 1200 rpm for 5 min to remove excess antibodies. Cell pellets were resuspended in 500 μl FACS buffer and passed through a 40 μm cell strainer prior to FACS with the unstained and isotype-stained hESC-derived lung progenitors as negative controls for gating parameters.

### Air-liquid interface culture

150,000 Day 15-sorted CD166+ cells were seeded onto matrigel-coated 6.5 mm transwell inserts and cultured in basal medium containing 250 ng/ml FGF2, 100 ng/ml FGF10, 50 nM dexamethasone (Sigma-Aldrich), 0.1 mM 8-bromoadenosine 30,50-cyclic monophosphate sodium salt (cAMP, Sigma-Aldrich) and 0.1 mM 3-Isobutyl-1-methylxanthine (IBMX) (Sigma-Aldrich) for 2 weeks. The cell culture medium was refreshed every other day. At Day 31, air-liquid interface culture was initiated by removing medium from the apical chamber and adding PneumaCult medium supplemented with 10 µM DAPT to the basal chamber. The cell culture medium in the basal chamber was refreshed every other day until Day 45.

### Virus and related cell cultures

All influenza virus strains A/Puerto Rico/8/1934, A/WSN/1933, and A/Aichi/2/1968 were purchased from the American Type Culture Collection (ATCC) propagated in eggs and titrated using plaque assays with Madin-Darby canine kidney cells (ATCC) as described below. MDCK cells were cultured in Eagle's minimal essential medium supplemented with 10% fetal bovine serum (FBS).

### Infection of hPSC-derived airway epithelial cells with influenza virus H1N1 (A/Puerto Rico/8/1934 & A/WSN/1933) and H3N2 (A/Aichi/2/1968)

hPSC-derived airway epithelial cells cultured on transwell inserts were washed once with 50 μl of DPBS at 37°C for 10 min. All virus strains were thawed on ice and immediately diluted in 100 ml of PneumaCult ALI Maintenance medium (STEMCELL Technologies) with a multiplicity of infection (MOI) of 0.1. Total cell number was estimated by trypsinisation of hPSC-derived airway epithelial cells using a haemocytometer. DPBS was removed and replaced with virus inoculums in the apical chamber of the transwells and incubated at 35°C with 5% CO_2_ for 1 h. ‘Clean’ wells did not contain any medium. PneumaCult ALI Maintenance medium, without either virus strains, was added as the ‘mock infection control’ well. Inoculums were removed from the apical chambers, and the transwells were transferred into a fresh 24-well plate, with basal chambers containing 350 μl of PneumaCult ALI Maintenance medium. The cells were then incubated at 37°C with 5% CO_2_ for 24 h. 50 μl of PBS was then incubated in the apical chamber for 10 min at 37°C with 5% CO_2_ to collect progeny viruses.

### Virus plaque assay

MDCK cells were cultured in 24-well plates to 95% confluency. Cells were then incubated with 100 ml of serial diluted (10^1^ to 10^6^), trypsin-TPCK (1 mg/ml) activated virus samples derived from infected hPSC-derived airway epithelial cell culture medium at 35°C with 5% CO_2_ for 1 h. Plates were gently rocked every 15 min to allow for an equal distribution of the virus samples. Inoculums were removed and replaced with 1 ml of 1.2% Avicel overlay per well. Cells were incubated at 35°C with 5% CO_2_ for 65 to 72 h. The Avicel overlay was aspirated, and cells were then fixed with 500 μl of 4% formaldehyde in PBS for 1 h. Formaldehyde was removed, and cells were gently washed with PBS. Fixed cells were stained with 1% crystal violet for a maximum of 15 min and gently washed with running water. Plates were air-dried at room temperature (RT) and plaque forming units (PFU) were calculated as follows: number of plaques×dilution factors=number of PFU per 100 μl (Qiao et al., 2018).

### Immunofluorescence

Cells were washed with DPBS and then fixed in 4% PFA for 20 min at RT. The PFA was removed, and cells were washed with DPBS before they were blocked and permeabilized with 10% donkey serum in 0.1% Triton X-100 in DPBS for 1 h at RT. Primary antibodies diluted in 1% donkey serum in DPBS were applied to the cells and left to incubate overnight at 4°C. Excess primary antibodies were then removed, and cells were washed three times with DPBS. Cells were incubated with secondary antibodies diluted in 1% donkey serum in DPBS for 1 h at RT in the dark. Cells were then washed three times with DPBS before they were incubated with Hoescht diluted in DPBS for 5 min at RT in the dark to allow visualization of nuclei. Images were captured using an Olympus Fluoview FV1000 confocal microscope and analysed using ImageJ. Primary and secondary antibodies used in this study can be found in Table S2.

### RNA extraction, cDNA synthesis and quantitative real-time PCR (qPCR)

RNeasy kit (Qiagen) was used to extract total RNA as per manufacturer's instructions. The High-Capacity cDNA Reverse Transcription Kit (Applied Biosystems) was used to convert 500 ng of purified RNA into cDNA. qPCR reactions were run on the QuantStudio 7 Flex Real-Time PCR System. Samples were run in duplicates and normalized to the housekeeping gene b ACTIN. Sequences of primers used can be found in Table S1. Adult lung mRNA was obtained from Biochain (Total RNA – human adult normal tissue: lung lower left lobe; catalogue number #R1234152-50; lot #B6050780).

### Cell lysis and western blotting

Similar to previous studies (49, 50) protein lysates of the hPSC-derived airway epithelial cells were collected using Triton X-100-based protein lysis buffer supplemented with protease inhibitors. Following protein content determination using the Bio-Rad protein assay kit (Bio-Rad, USA), lysates were prepared with β-mercaptoethanol and resolved using SDS-PAGE then transferred onto PVDF membranes prior to incubation with the relevant primary antibodies (Table S2). Membranes were then incubated with respective secondary antibodies followed by visualization via enhanced chemiluminescence.

### Statistical analyses

GraphPad Prism 8.0 (GraphPad Software Inc., San Diego, CA, USA) was used to plot all graphs and perform all statistical analyses. Error bars indicate mean±standard deviation (SD). All experiments were performed with at least 3 biological replicates. One-way Analysis of Variance (ANOVA) followed by Tukey's multiple comparisons analysis or multiple comparisons with Dunnett's correction was used to determine statistical significance. *P* values of <0.05 were considered statistically significant.

## Supplementary Material

10.1242/biolopen.061729_sup1 Supplementary information
