# Supplementary material for: Differentiation of CD166-positive hPSC-derived lung progenitors into airway epithelial cells
Source: Biol Open. 2024 Oct 10;13(10):bio061729. doi: 10.1242/bio.061729 (PMC11554259; doi:10.1242/bio.061729)
Supplement: Supplementary information [file biolopen-13-061729-s1.pdf]

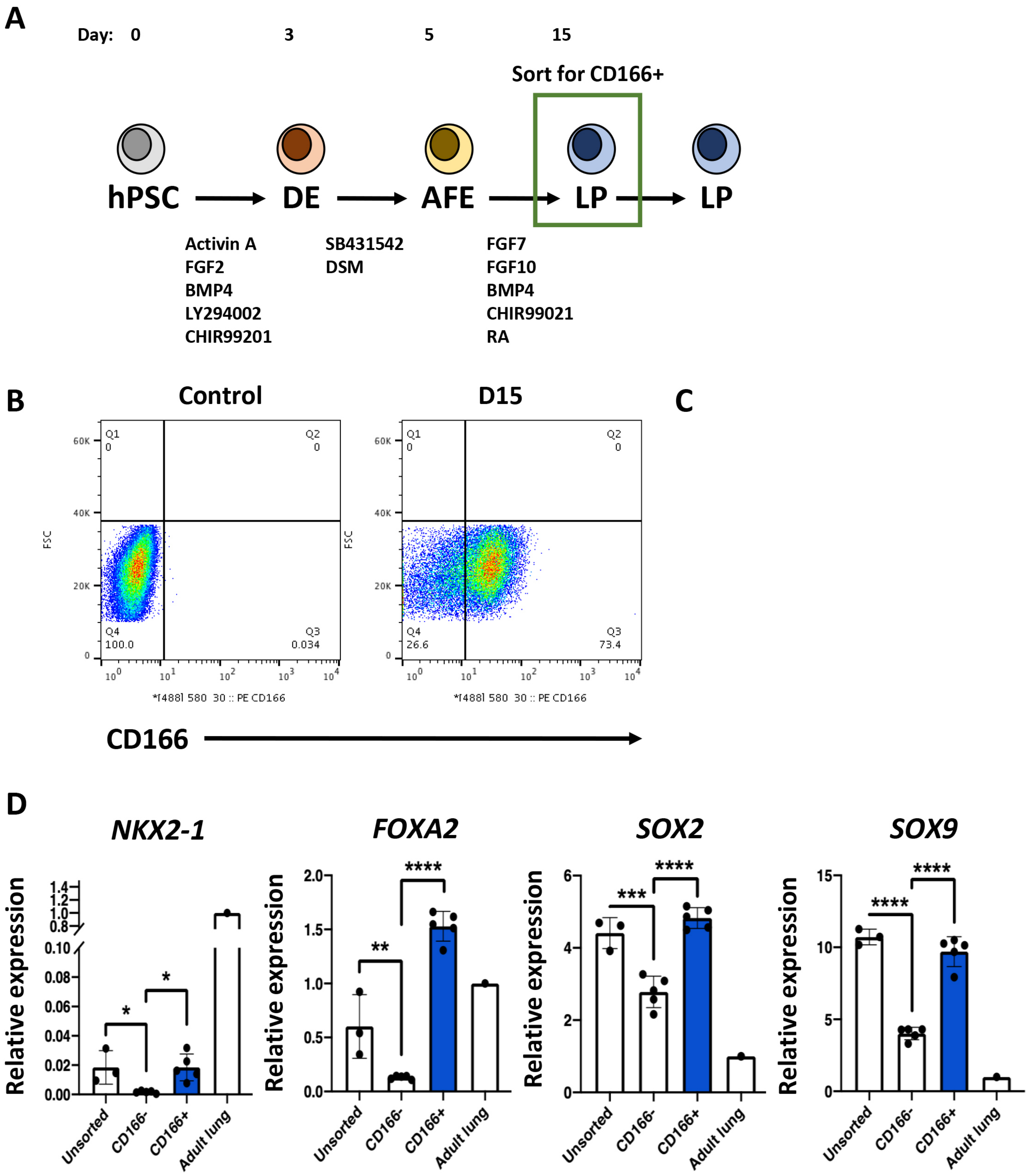

Fig. S1. Early lung progenitors can be isolated from heterogeneous differentiation cultures using the cell surface marker CD166.

(A) Schematic showing differentiation of human pluripotent stem cells (hPSC) into definitive endoderm (DE), anterior foregut endoderm (AFE) and finally into lung progenitors (LPs) that can be purified by sorting for CD166+ cells.

(B) Representative dot plot of Day 15 cells sorted for CD166.

(C) Quantification of CD166+ cell numbers at Day 15. n=7.

(D) qPCR analysis of early lung progenitor markers *NKX2-1, FOXA2, SOX2* and *SOX9*. Data from at least 3 independent experiments presented as means ± s.d. *p<0.05, **p<0.01, ****p<0.0001.

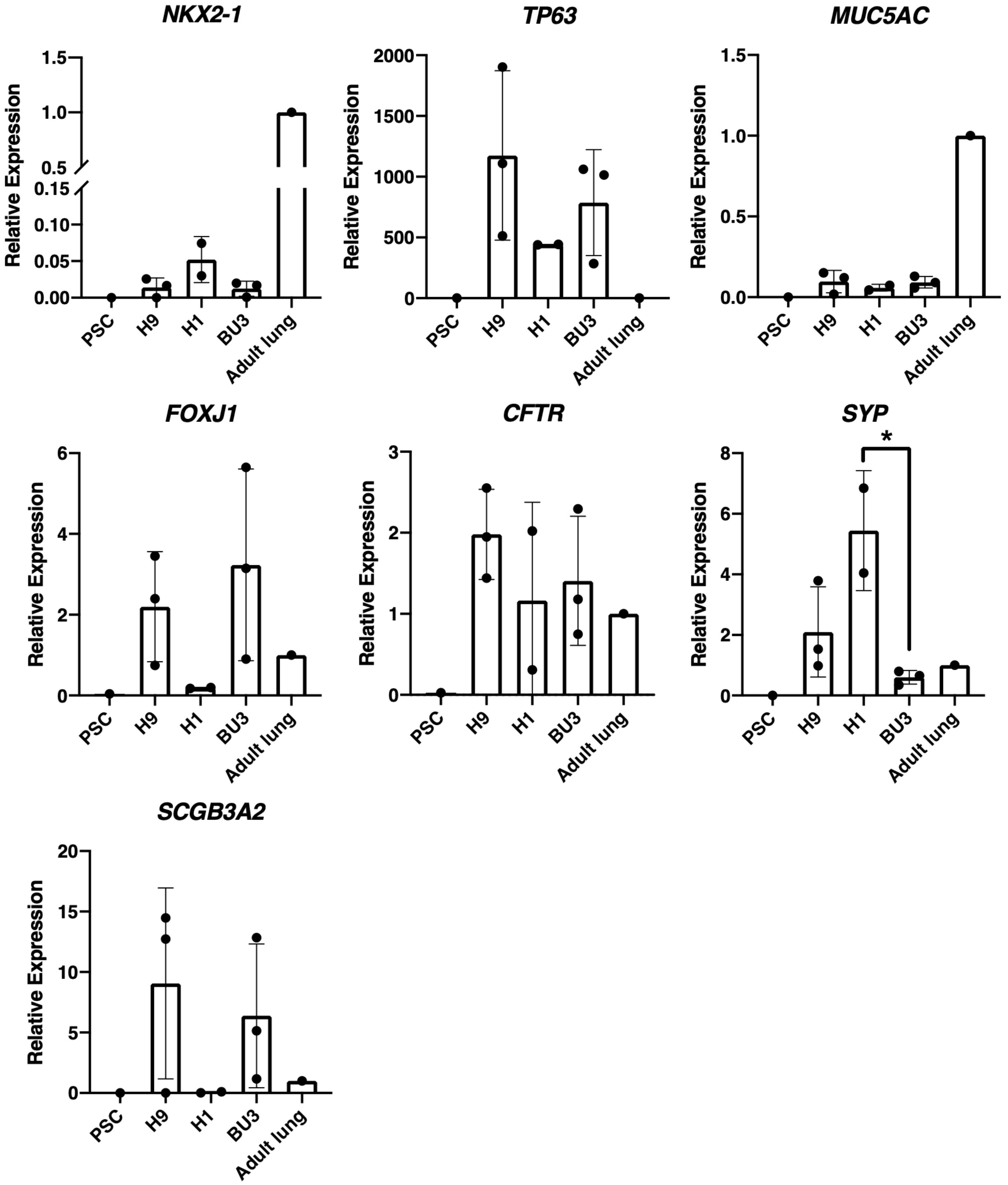

Fig. S2. H1 ESCs and BU3 *NKX2-1*^{eGFP} iPSCs can be differentiated into proximal airway epithelial cells. qPCR analysis of proximal airway markers *NKX2-1*, *TP63*, *MUC5AC*, *FOXJ1*, *CFTR*, *SYP* and *SCGB3A2*. Data generated from at least 2 independent experiments presented as means ± s.d. *$p<0.05$. PSC: pluripotent stem cell.

Biology Open • Supplementary information

Table S1. List of qPCR primer sequences

Name	Forward Sequence (5’ → 3’)	Reverse sequence (5’ → 3’)
ACTB	CTGGAACGGTGAAGGTGACA	AAGGGACTTCCTGTAACAATGCA
CFTR	CTATGACCCGGATAACAAGGAGG	CAAAAATGGCTGGGTGTAGGA
FOXA2	AGGAGGAAAACGGGAAAGAA	CAACAACAGCAATGGAGGAG
FOXJ1	CACGTGAAGCCTCCCTACTC	GGATTGAATTCTGCCAGGTG
MUC5AC	CCATTGCTATTATGCCCTGTGT	TGGTGGACGGACAGTCACT
NKX2-1	ACCAGGACACCATGAGGAAC	CGCCGACAGGTACTTCTGTT
SCGB3A2	CAAGTGGAACCACTGGCTTG	CCAGAGGTAAAGGTGCCAAC
SOX2	TGGACAGTTACGCGCACAT	CGAGTAGGACATGCTGTAGGT
SOX9	GAACGCACATCAAGACGGAG	AGTTCTGGTGGTCGGTGTAG
SYP	TTTGTGAAGGTGCTGCAATG	ACCTCGATGCTGAGGTCACT
TP63	AAAGACATGCCCCATCCAGA	CATACTGGGCATGGCTGTTC

Biology Open • Supplementary information

Table S2. List of antibodies

**Primary antibodies**					
**Name**	**Species**	**Application**	**Company**	**Catalogue no./reference**	**Dilution**
ALCAM Phycoerythrin MAb (Clone 105902)	Mouse	Flow cytometry	R&D Systems	FAB6561P	1:200
Anti-p63 antibody [Y289]	Rabbit	IF	Abcam	ab32353	1:100
Anti-Cytokeratin 5 antibody [XM26]	Mouse	IF	Abcam	ab17130	1:100
SPLUNC1 antibody	Goat	IF	Kind gift from C. D. Bingle at the Academic Unit of Respiratory Medicine, Department of Infection, Immunity and Cardiovascu-lar Disease, University of Sheffield, UK	Bingle et al. *J. Pathol*. 2005 205:491-7	1:300
SCGB1A1 antibody	Rabbit	IF	Abcam	ab40873	1:200
Anti-Acetylated tubulin antibody	Mouse	IF	Sigma	T6793	1:500
Nucleoprotein (NP)	Rabbit	WB	Abcam	AB104870	1:1000
Nonstructural protein 1 (NS1)	Rabbit	WB	Thermo-fisher Scientific, Rockford, Ill	PA5-32243	1:1000
Matrix protein 1 (M1)	Rabbit	WB	Thermo-fisher Scientific	PA5-32222	1:1000
β-tubulin	Mouse	WB	Proteintech	66240-1-Ig	1:10000
**Secondary antibodies**					
**Target protein**	**Species**	**Application**	**Company**	**Catalogue No.**	**Dilution**
Anti-Rabbit IgG (H+L), Alexa Fluor 546	Donkey	IF	Invitrogen	A10040	1:1000
Anti-Mouse IgG (H+L), Alexa Fluor Plus 488	Donkey	IF	Invitrogen	A32766	1:1000

Biology Open • Supplementary information
